# Mechanics Underpinning Phase Separation of Hydrogels

**DOI:** 10.1021/acs.macromol.2c02356

**Published:** 2023-01-05

**Authors:** Yu Zhou, Lihua Jin

**Affiliations:** Department of Mechanical and Aerospace Engineering, University of California, Los Angeles, Los Angeles, California90095, United States

## Abstract

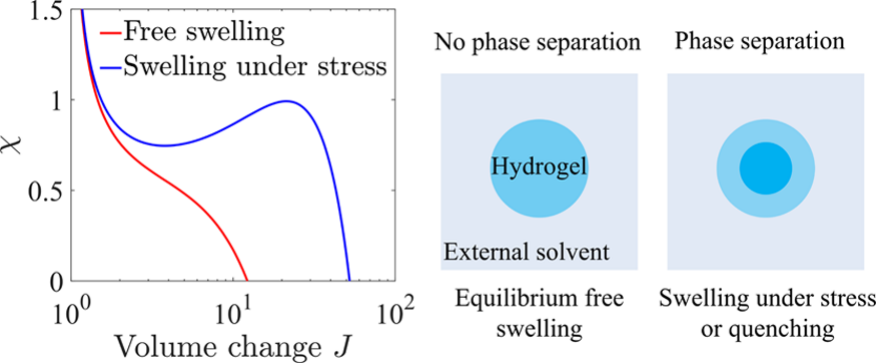

This paper reveals the underpinning role of mechanical constraints and dynamic loading in triggering volume phase transitions and phase separation of hydrogels. Using the Flory–Rehner free energy that does not predict phase separation of hydrogels under equilibrium free swelling, we show that mechanical constraints can lead to coexistence of multiple phases. We systematically obtain the states of equilibrium for hydrogels under various mechanical constraints and unravel how mechanical constraints change the convexity of the free energy and monotonicity of the stress–stretch curves, leading to phase coexistence. Using a phase-field model, we predict the pattern evolution of phase coexistence and show that many features cannot be captured by the homogeneous states of equilibrium due to large mismatch stretch between the coexisting phases. We further reveal that the system size, quenching rate, and loading rate can significantly influence the phase behavior, which provides insights for experimental studies related to morphological patterns of hydrogels.

## 1 Introduction

A hydrogel is composed of a polymer network dispersed in a solvent, and its volume can significantly change through the transport of solvent molecules. In response to small changes of external stimuli, such as the temperature,^1^ solvent composition,^2^ electric field,^3^ and light,^4^ certain hydrogels can undergo a discontinuous volume transition from a swollen state to a shrunk state, which is called a volume phase transition.^5−7^ Hydrogels capable of a volume phase transition have diverse applications, including sensors,^8^ actuators,^9^ soft robots,^10^ drug delivery,^11^ and so on. During the process of a volume phase transition, which is governed by the kinetics of solvent migration, phase separation occurs, and the swollen and shrunk states coexist. Phase separation has been utilized to enhance the mechanical properties, such as stiffness and toughness, of hydrogels.^12−17^ For example, a soft poly(acrylic acid) hydrogel containing calcium acetate, which helps form hydrophobic complexes, can be rapidly switched to a rigid plastic due to phase separation via spinodal decomposition with its volume almost conserved, leading to tremendous enhancement of stiffness and strength.^18^

Volume phase transitions and phase separation of hydrogels have been experimentally demonstrated in various hydrogels.^19^ Tanaka et al. found that neutral polyacrylamide (PAAm) hydrogels immersed in a basic solution can become polyelectrolyte hydrogels via hydrolysis.^2,20^ While neutral PAAm hydrogels undergo a continuous volume change with the acetone concentration, the resultant polyelectrolyte hydrogels in an acetone solution can undergo a discontinuous volume transition by changing the acetone concentration, which is attributed to the osmotic pressure of dissociated hydrogen ions.^2^ Hirokawa and Tanaka reported that neutral poly(*N*-isopropylacrylamide) (PNIPAM) hydrogels immersed in water could also exhibit a volume phase transition by changing temperature.^21^ Shen et al. demonstrated that blisters form on the surface of neutral PNIPAM hydrogels when they are subjected to heating, where an impermeable skin layer is formed during phase separation, and water fills into the defects under the skin layer, forming inflated blisters.^22^ When subjected to heating, a neutral PNIPAM hydrogel cylinder shows coexistence of swollen and shrunk phases along the radial direction, while a weakly ionized PNIPAM hydrogel cylinder shows phase coexistence along the axial direction.^23,24^

Most theoretical studies on the volume phase transition of hydrogels are based on double-well free energy. Cai and Suo successfully demonstrated a volume phase transition of thermo-responsive hydrogels by obtaining the state of equilibrium by minimizing a properly constructed free energy function and predicted phase coexistence based on the assumption of a sharp interface between the swollen and shrunk phases.^25^ Continuum field theory was developed by Yu et al. to predict the volume phase transition of polyelectrolyte hydrogels induced by changing the salt concentration of an external solution.^26^ The above-mentioned models are based on Flory–Rehner theory, where the Flory–Huggins interaction parameter χ, describing the interaction between a polymer and a solvent, is modeled as a function of the polymer volume fraction to obtain double-well free energy, thus, to capture the phase coexistence and the discontinuous volume change.^5^ Considering a sharp interface between the swollen and shrunk phases of a one-dimensional cylindrical or spherical hydrogel, Tomari and Doi numerically studied the kinetic process of the hydrogel undergoing a volume phase transition.^27−29^ By the introduction of interfacial energy and kinetics of diffusion, a phase-field model was developed to simulate the evolution of different phases and interfaces under two-dimensional deformation using a double-well free energy.^30^ Bao et al. utilized the phase-field model with proposed new free energy including the processes of detachment of polymer chains from and reattachment onto cross-links to simulate the three-dimensional morphology evolution of nanocomposite hydrogels during a phase separation process.^31^

Of particular interest is the influence of mechanical constraints on phase transitions and phase separation of hydrogels. Although it is well known that PAAm hydrogels do not have volume phase transitions under free swelling, Matsuo and Tanaka found that subjected to different axial stretches, PAAm hydrogels immersed in an acetone solution show different shapes and phase separation patterns.^32^ In particular, an impermeable skin layer forms on the surface of a PAAm hydrogel cylinder, which provides extra mechanical constraints.^32^ Based on the Flory–Rehner model with a constant Flory–Huggins interaction parameter χ, Hennessy et al. studied phase separation of a hydrogel under constrained uniaxial deformation, i.e., free deformation in one dimension but constrained in the other two dimensions. They found that a volume phase transition cannot occur when the hydrogel is in equilibrium with a solvent of zero chemical potential, but two phases can coexist when the hydrogel is in equilibrium with a solvent of higher chemical potential.^33,34^ Cirillo et al. predicted that when a hydrogel is subjected to a pressure load, swollen and shrunk phases only coexist under constrained uniaxial deformation, instead of constrained biaxial or three-dimensional volumetric deformation.^35^ Contradictorily, Duda et al. theoretically showed that hydrogels under hydrostatic loading could have multiphase equilibria, which indicates the possibility of a volume phase transition.^36^ Yamamoto et al. predicted that composite hydrogels with cofacially aligned nanosheets could undergo discontinuous deformation with a constant volume.^37−39^ The above-mentioned models are based on the Flory–Rehner free energy with the parameter χ independent of the polymer concentration, which is not double-well, and consequently, no volume phase transition can be predicted under free swelling. However, the introduction of mechanical constraints changes the total free energy, leading to possible phase coexistence. It is worth mentioning that Dušková-Smrčková and Dušek showed that the volume phase transition can be either induced or suppressed by expansive and compressive strain.^40^ Although the above-mentioned theoretical studies based on the Flory–Rehner model with a constant Flory–Huggins interaction parameter χ show possible phase transitions of hydrogels under certain constraints,^33,35,36^ they focus on the equilibrium states in one specific stress state or chemical potential loading, and some of the results seem even contradictory. This paper will systematically investigate the effect of mechanical constraints on the equilibrium states based on single-well Flory–Rehner free energy. We will not only identify the conditions for existence of multiple homogeneous equilibrium states but also use the phase-field model to demonstrate the pattern evolution of phase coexistence. Our results will show that the predicted multiple homogeneous equilibrium states cannot always well represent the swollen and shrunk phases in phase separation, attributed to the large stretch mismatch between the two separated phases.

Phase separation of hydrogels is limited by the diffusion process of solvent molecules, and therefore, loading rates can also play an important role in the phase behavior. When subjected to different heating rates, PNIPAM hydrogels could show different phase morphologies, including the fine and coarse surface patterns and the bubble pattern.^23,24^ Chang et al. demonstrated that a toroid-shaped PNIPAM hydrogel subjected to slow heating maintains toroidal shape, while the hydrogel subjected to rapid heating buckles and undergoes mechanical instability.^41^ Considering the kinetics of diffusion, Hennessy et al. have shown that temporary phase separation can occur in a neutral hydrogel under free swelling by quenching it through a sudden increase in the interaction parameter χ.^33^ However, how a quenching rate, which is different from the diffusion time scale, affects the texture and morphology of phase-separated hydrogels still needs to be investigated. In addition, when a hydrogel is mechanically loaded, the influence of stretching rates on its phase separation is yet to be explored. To address the above-mentioned questions, we utilize the phase-field modeling to demonstrate phase separation and coexistence of hydrogels under different mechanical constraints and loading conditions. The influence of the system size, stretching rate, and quenching rate on the volume phase transition and phase separation of hydrogels will be studied.

In this paper, we first briefly introduce the constitutive models of hydrogels in Section 2. We then obtain homogeneous solutions and the onset conditions of phase separation of hydrogels under free swelling, hydrostatic loading, constrained uniaxial deformation, and constrained biaxial deformation in Section 3. Using the phase-field model, we simulate the phase separation and pattern evolution of hydrogels under one-dimensional and two-dimensional settings with finite system sizes in Section 4. We further investigate the effect of quenching and stretching rates in Section 5. Section 6 concludes the paper.

## 2 Constitutive Models of Hydrogels

**Figure 1:**
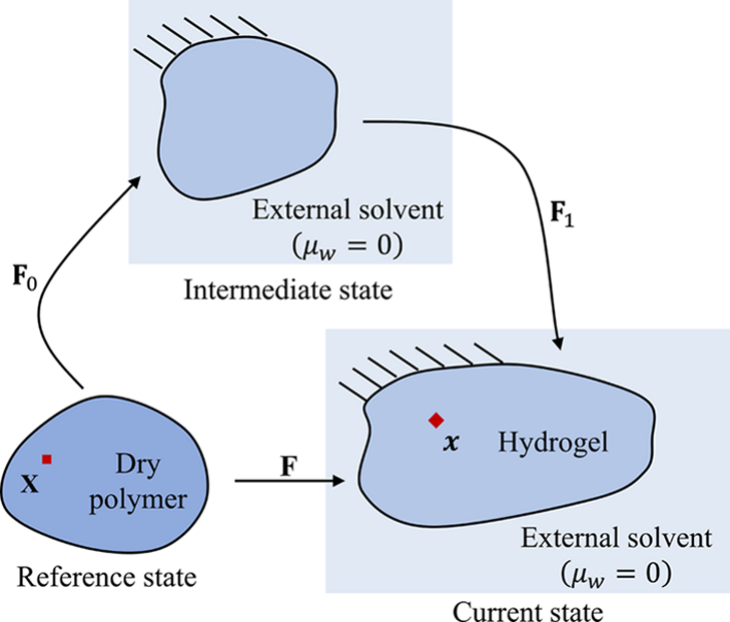
Schematic of the reference, intermediate, and current states. The intermediate state is chosen as the initial state in numerical simulations.

Here, we consider the deformation of a hydrogel subjected to applied mechanical loadings and solvent exchanges with an external solvent. The dry polymer is taken as the reference state, where the material point of the hydrogel labeled with a coordinate ***X*** moves to a place with a coordinate ***x*** in the current state at time *t* (Figure 1). The deformation gradient tensor of the hydrogel is defined as **F** = ∂***x***/∂***X*** or , whose determinant *J* = det **F** represents the volume change of the material point. The number of solvent molecules per unit reference volume is denoted *C*. The polymer network and solvent are assumed to be incompressible, which gives
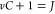
1where *v* is the volume of a solvent molecule. The inverse of the swelling ratio is related to the polymer concentration ϕ via 1/*J* = ϕ.

The free energy of the hydrogel per unit reference volume is contributed by the stretching of the network and the mixing of the polymer and solvent^42−44^
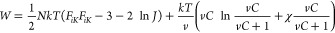
2where *N* is the cross-link density of the hydrogel in the dry state, *k* is the Boltzmann constant, *T* is the temperature, and χ is the interaction constant measuring the enthalpy of mixing between the polymer and solvent. The parameter χ is often a function of polymer concentration χ = χ(ϕ), empirically fitted from experimental results.^40,45^ Specifically, χ often increases with ϕ for poor solvents, while it is nearly independent of ϕ for good solvents.^46^ There is not a universal χ(ϕ) function. Although a frequently used form is a polynomial relation, the fitted parameters can vary widely. Here, our goal is to demonstrate that mechanical constraints can lead to coexistence of multiple phases in hydrogels where coexistence does not occur without constraints. The Flory–Rehner free energy with the parameter χ independent of ϕ describes such hydrogels, and it does not predict volume phase transitions of these hydrogels under equilibrium free swelling. Therefore, instead of choosing a specific χ(ϕ) function with a specific set of parameters, here, we simply assume that χ is independent of ϕ for the majority of the demonstration in this paper. The dependence of χ on other external fields, such as temperature χ = χ(ϕ), is directly prescribed as variations of χ within a reasonable range from 0 to 2, comparable to that in the literature.^37,40^ To further show that our method can be easily extended to different χ(ϕ) functions, in the Supporting Information, we demonstrate the phase behavior of a hydrogel with χ = χ_1_ + χ_2_ϕ as an example. It is worth mentioning that there are many other forms of stretching energy considered in the literature, and the stretching energy can also be formulated by choosing the initial fabrication state as the relaxation state of the polymer network,^7,47−49^ the energy which tends to become double-welled more easily under mechanical constraints. Therefore, here, we show that even with the current stretching energy in (2), a hydrogel with a single phase under equilibrium swelling can undergo complex phase behaviors under mechanical constraints. Our method and results can be easily extended to hydrogels with other forms of free energy.

Given the free energy, the nominal stress can be obtained as^50^
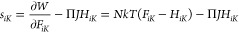
3where Π is a Lagrange multiplier to enforce the incompressibility condition (1) and *H*_*iK*_ defined by  leads to **H** = **F**^–*T*^. The chemical potential of the solvent molecules in the hydrogel can be calculated as^50^
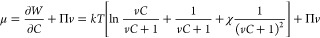
4

When the hydrogel reaches chemical equilibrium, the chemical potential μ equals the chemical potential of the solvent in the environment, μ_w_. We assume μ_w_ = 0, unless otherwise mentioned. Several cases of homogeneous deformation are studied in the next section.

## 3 Homogeneous State of Equilibrium

In this section, we study homogeneous deformation of a hydrogel, its energy landscape, and stability under various mechanical constraints. A local minimum of free energy corresponds to an equilibrium state of the hydrogel. Therefore, the coexistence of multiple phases is identified by the existence of multiple local minima and by analyzing the non-convexity of the free energy.

We consider a hydrogel immersed in a solvent subjected to a certain mechanical constraint. Four cases are studied here: (1) free swelling, (2) swelling under hydrostatic loading, (3) swelling with constrained uniaxial deformation, and (4) swelling with constrained biaxial deformation. For case (1), no mechanical constraint is applied on the hydrogel, and the swelling is assumed to be isotropic so that *F*_*iK*_ = λδ_*iK*_ = *J*^1/3^δ_*iK*_, where λ is the stretch in all directions. For case (2), the hydrogel is subjected to a hydrostatic loading with a fixed nominal stress *s*. The swelling is assumed to be isotropic, *F*_*iK*_ = λδ_*iK*_. It is noted that this hydrostatic loading defined by fixed nominal stress is different from the usual hydrostatic stress defined by fixed true stress. For case (3), two principal stretches of the hydrogel are assumed to be one, and in the other principal direction, the hydrogel exerts a nominal stress *s*. The deformation gradient can be written as **F** = diag(λ,1,1). For case (4), the hydrogel is assumed to undergo isotropic in-plane deformation with fixed equi-biaxial nominal stress *s*. The other principal stretch is assumed to be one. The deformation gradient can be written as **F** = diag(λ,λ,1).

Combined with the incompressibility constraint (1), the free energy (2) becomes
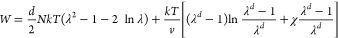
5where *d*(=1,2,3) represents the dimension of deformation. *d* = 1 corresponds to case (3), *d* = 2 corresponds to case (4), and *d* = 3 corresponds to cases (1) and (2). Selecting the system to be the hydrogel, the environment, and the load, we obtain the total free energy of the system to be the sum of that of all the components
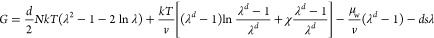
6

When the system is in equilibrium, the first derivative of the free energy *G* with respect to λ vanishes
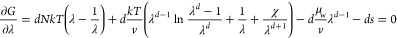
7

The stability criterion for the system is determined by the second derivative of *G* with respect to λ
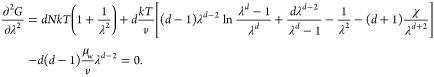
8

When ∂^2^*G*/∂λ^2^ > 0, the system is stable.

### 3.1 Free Swelling

When the hydrogel is under free swelling, we have *s* = 0. Equations 7 and 8 determine the condition of equilibrium and the stability of the system. For the free swelling case, we use the swelling ratio *J* as a variable and obtain the equivalent forms for eqs 7 and 8. The free energy (2) expressed as a function of *J* is
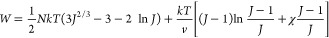
9

When the hydrogel is in equilibrium, eq 7 is equivalent to the following equation
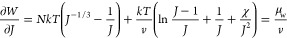
10

**Figure 2:**
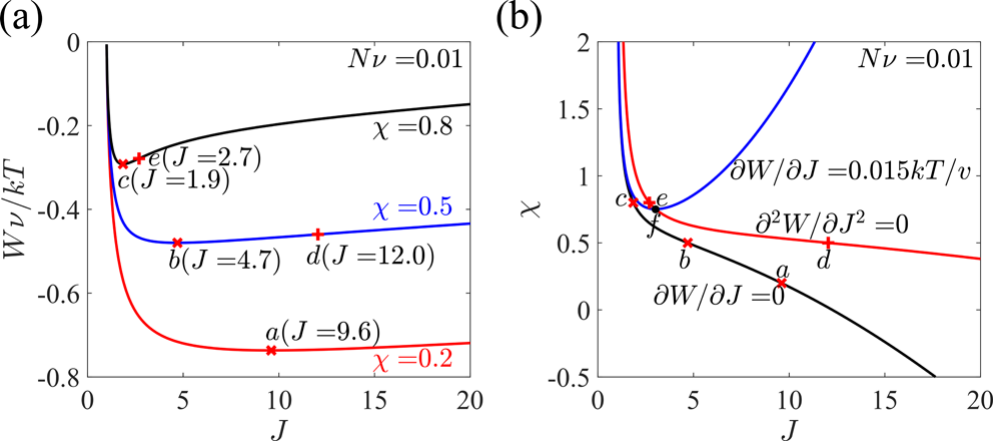
A hydrogel is in equilibrium with an external solvent without mechanical constraints. (a) Free energy as a function of the swelling ratio *J* at various χ. Points *a*, *b*, and *c* correspond to the equilibrium solutions when μ_w_ = 0, and points *d* and *e* are the solutions to eq 11. (b) Equilibrium solutions under μ_w_ = 0 (the black curve) and μ_w_ = 0.15*kT* (the blue curve) and the spinodal curve (the red curve). Points *a*–*e* correspond to the same conditions as those in (a), while point *f* is the intersection of solutions to eq 10 (i.e., the equilibrium solutions) and to eq 11 when μ_w_ = 0.015*kT*.

The free energy *W* is plotted as a function of *J* at different values of χ, and points *a*, *b*, and *c* correspond to the equilibrium solutions when μ_w_ = 0 (Figure 2a).

Whether the system is stable or not can also be determined by the second derivative of *W* with respect to *J*
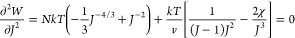
11

Equation 11 is equivalent to eq 8, if μ_w_ is eliminated by substituting eq 7 into 8. Equation 11 determines the stability of the system regardless of the value of μ_w_. When ∂^2^*W*/∂*J*^2^ is positive, the hydrogel prefers to stay homogeneous, while when ∂^2^*W*/∂*J*^2^ is negative, phase separation occurs. The solutions to eq 11 are shown as points *d* and *e* in Figure 2a, where we have ∂^2^*W*/∂*J*^2^ > 0 when the swelling ratio is smaller than that of point *d* or *e*. When χ = 0.2, the solution to eq 11 is *J* = 29.3, which is not shown in Figure 2a. Clearly, when μ_w_ = 0, all three equilibrium solutions *a*, *b*, and *c* are stable.

Solutions to eq 10 under μ_w_ = 0 (the black curve) and μ_w_ = 0.015*kT* (the blue curve) and to eq 11 (the red curve) are plotted as functions of χ (Figure 2b). The red curve, corresponding to the solution to eq 11, is called the spinodal curve. Above the curve, we have ∂^2^*W*/∂*J*^2^ < 0, and the system is unstable. The black curve of the equilibrium solutions when μ_w_ = 0 is always below the spinodal curve, indicating that the equilibrium states are stable. The blue curve of the equilibrium solutions when μ_w_ = 0.015*kT* intersects with the red curve at point *f* corresponding to *J* = 3.02 and χ = 0.75. Under a given χ larger than 0.75, there are two solutions of *J* to eq 10. The solution corresponding to *J* smaller than 3.02 is stable since it is below the red curve, while the other solution is unstable. In general, for μ_w_ > 0, there are either zero, one, or two solutions to eq 10 for a given χ, where one solution corresponds to the intersection of solutions to eqs 10 and 11, for example, point *f* for μ_w_ = 0.015*kT*. When there are two solutions, one solution is stable, and the other one is unstable. Since there is at most one stable solution, there is no phase coexistence when the hydrogel is under equilibrium. According to the literature and the discussion of Figure S1 in the Supporting Information, when the parameter χ depends on the swelling ratio *J*, there might exist two stable equilibrium solutions.^25^ As a conclusion, although phase coexistence can be predicted when the parameter χ depends on the swelling ratio *J*, for the case of free swelling, the free energy (9) with constant χ cannot predict equilibrium phase coexistence.

### 3.2 Swelling under Hydrostatic Loading

**Figure 3:**
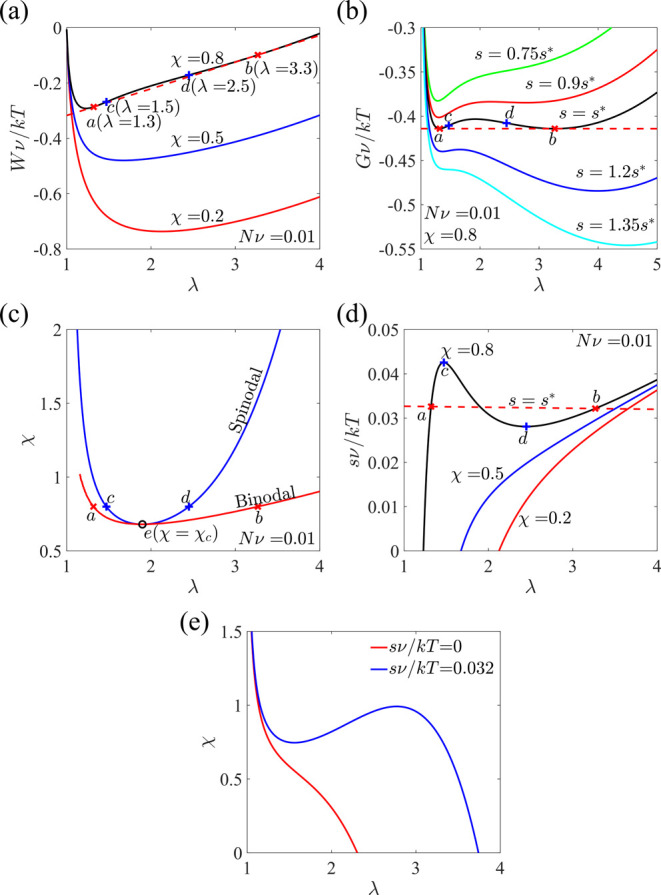
A hydrogel subjected to hydrostatic loading is in equilibrium with an external solvent. (a) Free energy *W* as a function of stretch at various χ. Points *a* and *b* are the two intersection points of the *W*–λ curve and the common tangent line (the red dashed line), and points *c* and *d* are solutions to eq 8. (b) Free energy *G* = *W* – 3*s*λ as a function of stretch for χ = 0.8 at different values of *s*. (c) Spinodal and binodal curves with their intersection point *e*. (d) Nominal stress as a function of stretch at various χ. Points *a*–*d* correspond to the same conditions as those in (a–d). (e) Equilibrium solutions under *s* = 0 (the red curve) and *s* = 0.032*kT*/*v* (the blue curve).

We next consider a hydrogel under a hydrostatic loading immersed in a solvent with μ_w_ = 0. The free energy *W* is plotted as a function of λ at different values of χ (Figure 3a). The convexity of the curve changes when χ increases. When χ is small, the free energy is convex for the entire range of stretch λ; the corresponding examples of χ = 0.2 and 0.5 are shown in Figure 3a. As eq 7 shows, the system reaches equilibrium when ∂*G*/∂λ = ∂*W*/∂λ – 3*s* = 0, where the slope of the free energy *W* is equal to 3*s*. Since ∂^2^*G*/∂λ^2^ = ∂^2^*W*/∂λ^2^ > 0 when χ is small, there is only one solution of stretch for a given external stress. On the other hand, when χ is large, the free energy becomes concave for an intermediate range of stretch λ. For example, for χ = 0.8, there is a common tangent line (the red dashed line in Figure 3a) intersecting the *W*–λ curve at points *a* and *b*. The slope of the common tangent 3s* corresponds to the critical stress s* for coexistence, when the free energy *G* for the coexisting phases is equal. We further plot the free energy of the system *G* = *W* – 3*s*λ defined in eq 6 as a function of λ in Figure 3b. When *s* = s*, the free energy *G* indeed has two local minima with equal values, indicating that the two phases can coexist when stress s* is applied. When *s* is slightly smaller (*s* = 0.9s*) or larger (*s* = 1.2s*) than s*, the free energy *G* is still double-well, but the shrunk phase has lower or higher free energy than the swollen phase, respectively. As *s* further decreases (*s* = 0.75s*) or increases (*s* = 1.35s*), the free energy *G* becomes single-well, with the single minimum corresponding to one stable equilibrium shrunk or swollen phase, respectively. The two local minima shown in Figure 3b correspond to the two tangent points shown in Figure 3a. Using the condition ∂^2^*G*/∂λ^2^ = 0, we identify points *c* and *d*. When a stretch is between points *c* and *d*, we have ∂^2^*G*/∂λ^2^ < 0, which indicates that the system is unstable, and phase separation might occur. Upon connecting all the tangent points for various χ, we obtain the binodal curve (the red curve in Figure 3c). The spinodal curve and the binodal curve merge at the critical point *e*, corresponding to a critical interaction parameter χ_*c*_ and a critical stretch λ_*c*_, where χ_*c*_ is the smallest value of χ that can induce phase separation. Solving eq 7, we plot the nominal stress *s* as a function of λ at different values of χ (Figure 3d). When χ is smaller than χ_*c*_, the nominal stress increases monotonically as λ increases, and the corresponding free energy is convex (Figure 3a). When χ is larger than χ_*c*_, the stress–stretch curve becomes non-monotonic, and there might exist three stretch solutions at a fixed stress value. The non-monotonic stress–stretch curves are also plotted with χ = χ_1_ + χ_2_ϕ (Figure S1e). The volume phase transition might occur with two phases coexisting when the hydrogel is gradually stretched to a certain range. It is worth mentioning that the stretch for coexisting phases may not be predicted accurately. Taking χ = 0.8 as an example, if both coexisting phases follow the assumed deformation *F*_*iK*_ = λδ_*iK*_, the stretch values of the two phases correspond to those of points *a* and *b* in Figure 3, which minimizes the free energy of the system consisting of the hydrogel and the load. However, the two separated phases may not follow the assumed homogeneous deformation due to the stretch mismatch between the two phases. Therefore, the free energy *W* of the hydrogel may not be simply written as eq 5. The solution to this issue will be discussed in Section 4.

Figure 3e shows the equilibrium solutions under free swelling (the red curve) and hydrostatic loading (the blue curve). It is noted that the free swelling case (μ_w_ = 0 and *s* = 0) is a special case of swelling under hydrostatic loading when the applied stress equals zero. From Figure 3e, it is shown that when the stress equals zero, there exists only one solution of stretch for a given parameter χ, and the stretch decreases monotonically with the increase in χ. In contrast, when *s* = 0.032*kT*/*v*, the stretch changes non-monotonically with the increase in χ, and there might exist three solutions of stretch for given χ in a certain range of values, verifying the predictions of Duda et al.^36^ Meanwhile, Cirillo et al. predicted that when a hydrogel is subjected to a hydrostatic true stress, no phases coexist under three-dimensional volumetric deformation;^35^ in their study, the defined pressure load is equivalent to μ_w_ in our paper. According to our studies of hydrogels under free swelling, there is at most one stable solution for given μ_w_, which also validates the results of Cirillo et al. The seemingly contradictory results come from different mechanical loadings.

### 3.3 Swelling with Constrained Uniaxial and Biaxial Deformation

For a hydrogel with constrained uniaxial deformation, the free energy of the total system is
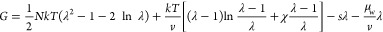
12

The equilibrium condition (7) can be rewritten as
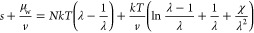
13

As clearly seen from eqs 12 and 13, the applied stress plays the same role as the chemical potential of the solvent in the equilibrium state for the constrained uniaxial case, which is not true for the constrained biaxial case.

**Figure 4:**
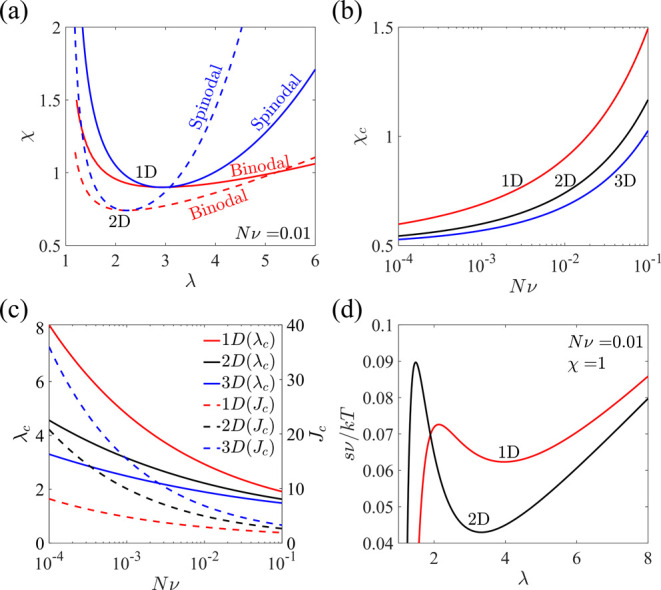
A hydrogel is in equilibrium with an external solvent under constrained uniaxial (1D) and biaxial (2D) deformation. (a) Spinodal and binodal curves. (b) Critical interaction parameter χ_*c*_ as a function of cross-link density *Nv* for constrained uniaxial, biaxial, and hydrostatic (3D) deformation. (c) Critical stretch λ_*c*_ and the corresponding critical volume *J*_*c*_ as functions of cross-link density *Nv*. (d) Non-monotonic nominal stress–stretch relations at high χ.

For both the constrained uniaxial and biaxial cases, the phase diagram under μ_w_ = 0 is shown in Figure 4a, where the solid curves correspond to the constrained uniaxial swelling, while the dashed curves correspond to the constrained biaxial case. At a fixed value of χ, there could exist multiple solutions of stretch λ. The critical interaction parameter χ_*c*_ of the biaxial case is lower than that of the uniaxial case, both of which are higher than that of the hydrostatic case (Figure 4a,b). This could be understood by the fact that lower-dimensional deformation means a higher degree of anisotropy of the deformation, which leads to higher elastic energy and requires higher χ_*c*_. χ_*c*_ increases with the increase in the normalized modulus *Nv*, which means that a poorer solvent is required to achieve phase separation when the network is more densely cross-linked (Figure 4b). Figure 4c shows that the critical stretch λ_*c*_ and the corresponding critical volume *J*_*c*_ decrease as *Nv* increases, while *J*_*c*_ increases, but λ_*c*_ decreases as the dimension of deformation increases (Figure 4c). When χ is beyond χ_*c*_, the stress–stretch curves are non-monotonic for both constrained uniaxial and biaxial cases (Figure 4d). Therefore, two phases might coexist for both cases.

It is worth mentioning that Cirillo et al. theoretically showed that two phases could coexist when a hydrogel is under constrained uniaxial deformation subjected to a fixed stress while the chemical potential is zero.^35^ Under similar constrained uniaxial deformation, Hennessy et al. also demonstrated that two phases could coexist when a hydrogel is prescribed with a fixed chemical potential while the stress is zero.^33^ Our equilibrium eq 13 shows the equivalent roles of applying a chemical potential and stress in phase separation of hydrogels with constrained uniaxial swelling. Kim et al. demonstrated that composite PNIPAM hydrogels with cofacially aligned nanosheets could rapidly respond to temperature changes.^39^ In their experiments, two neighboring paralleled nanosheets confine the deformation of the hydrogel in-between to be constrained uniaxial deformation. Parameter χ increases as temperature increases, leading to an increase in the chemical potential inside the hydrogel. According to our predictions, multiple equilibrium states can exist when a hydrogel with constrained uniaxial deformation is prescribed with a fixed chemical potential. Therefore, the hydrogel may transit from one state to another state, whose deformation is discontinuous and can occur rapidly. Our results could further justify the explanations of Yamamoto et al. on the experiments, who predicted that such constrained hydrogels can undergo discontinuous deformation with a constant volume.^37^

## 4 Numerical Simulations of Phase Separation

Our previous analysis is based on an assumption of homogeneous deformation. However, when a hydrogel is unstable and separates into two phases, it does not follow homogeneous deformation but highly inhomogeneous deformation with a large misfit between the two separated phases. In addition, the previous analysis is also not able to predict what the morphology of the separated phases is and how the phases evolve. To address these problems, the phase-field method is used to simulate the phase behaviors of hydrogels in this section.

### 4.1 Phase-Field Model

Selecting the concentration of solvent molecules *C* as the conservative phase-field variable, we introduce the gradient of the concentration to describe a diffuse interface between different phases. Following Hong and Wang, the free energy is assumed to be^30^
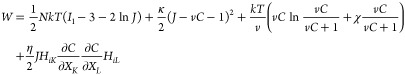
14where the second term is an energy penalty to enforce the incompressibility (1) with κ the bulk modulus and the last term is an ideal interface energy. The interfacial parameter η in eq 14 penalizes the formation of sharp interfaces and plays the role of surface tension. The mixing and interfacial energy defines a characteristic length . The mixing energy in eq 14 is different from the one used by Hong and Wang, where their mixing energy has double wells under free swelling when χ is large.^30^

By minimizing the total free energy of the system and combining the conservation of the solvent molecules, the stress equilibrium and the evolution equation of the phase field are obtained as^30^
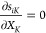
15
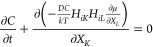
16where
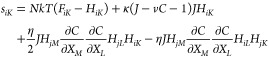
17
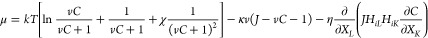
18

The Cauchy stress is related to the nominal stress by . The characteristic length *l* and the diffusivity *D* lead to a characteristic time . To solve the above-mentioned equations, chemical and mechanical boundary conditions are further needed. Assuming that the hydrogel is always immersed in a pure solvent, we have
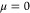
19at the interface between the hydrogel and the environmental solvent. Since the expression of chemical potential μ includes a second derivative of the concentration *C*, another boundary condition is needed. For all the boundaries, we require
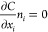
20where *n*_*i*_ is the unit outer normal of the boundaries in the current state. For the mechanical boundary conditions, either displacement or traction can be prescribed, which will be given in the following examples.

The weak form of eqs 15–18 is implemented into commercial software COMSOL to determine the temporal evolution of the fields of displacement, concentrations, and chemical potential. The cubic Lagrange element is used for the discretization of the displacement field, while the linear Lagrange element is used for the discretization of the concentration and chemical potential. The MUMPS solver and an implicit time discretization have been used for computation. Unless otherwise stated, a mesh size equal to the characteristic length has been used, achieving a good convergence of results. To enforce the incompressibility, κ = 1000*NkT* is chosen. Due to the singularity of the chemical potential in the dry state, an intermediate state with zero chemical potential is introduced as the initial state in numerical simulations (Figure 1). The total deformation gradient is decomposed as **F** = **F**_1_**F**_0_, where **F**_0_ maps the reference state to the intermediate state and **F**_1_ maps the intermediate state to the current state. The intermediate state is chosen to be homogeneous, and the initial stress obtained from eq 7 is applied to the hydrogel. In Sections 4.2 and 4.3, we start the simulations with a constant χ of a high value, and the hydrogels in the intermediate state are unstable. As a result, phase separation occurs, and swollen and shrunk phases evolve with time. To trigger the onset of the phase separation, a random small perturbation ranging from −5 × 10^–4^*C*_0_ to 5 × 10^–4^*C*_0_ is imposed on the initial concentration *C*_0_ for all simulations. The amplitude of the perturbation only influences the speed of the coarsening process in the early stage (*t* ∼ *l*^2^/*D*), and a slightly larger perturbation leads to faster initial coarsening. The initial small perturbation has little influence on the phase evolving process in the later stage; i.e., statistically equivalent patterns are obtained under different initial perturbations after *t* ∼ *l*^2^/*D*, and identical patterns are obtained after *t* ∼ *L*^2^/*D* with *L* the size of the hydrogel.

### 4.2 Phase Separation of Hydrogels under Constrained Uniaxial Deformation

**Figure 5:**
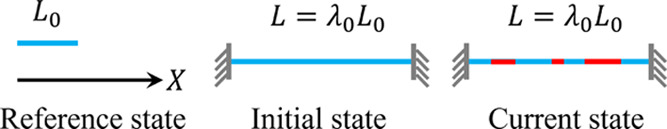
Schematic of a hydrogel under constrained uniaxial deformation in the reference, initial, and current states. In the current state, the hydrogel undergoes phase separation with different colors representing different stretch values of the swollen and shrunk phases.

We start from a hydrogel with two fixed ends and initial stretch λ_0_ = 3, where the length of the hydrogel in the initial state is denoted *L* (Figure 5). At the two ends of the hydrogel, we impose
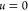
21where *u* is the displacement of the hydrogel in the current state with respect to the hydrogel in the initial state. As shown in Figure 4a, the hydrogel is unstable when the parameter χ is high, where phase separation is expected to occur. The total deformation gradient of the hydrogel with respect to the reference state is denoted **F** = diag(λ,1,1).

**Figure 6:**
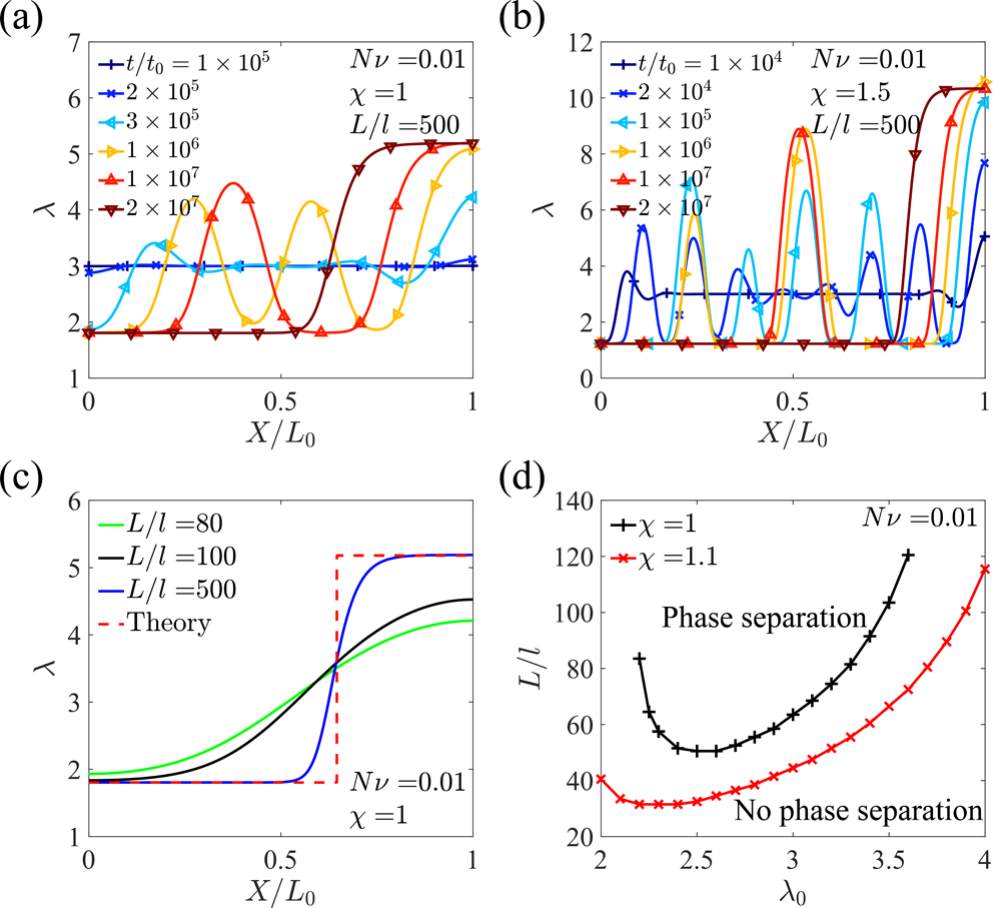
Temporal evolution of stretch as a function of the Lagrangian coordinate *X* associated with the reference state at (a) χ = 1 and (b) χ = 1.5. (c) Equilibrium distribution of stretch for hydrogels with different ratios of the sample length to the characteristic length, *L*/*l*. (d) *L*/*l*–λ_0_ diagram of phase separation at different χ.

The evolution of stretch as a function of the Lagrangian coordinate *X* associated with the reference state is shown in Figure 6a,b for different values of χ. The initial fluctuation grows and coarsens as a function of time. The domains near the boundary grow faster than those in the bulk because the gradient of the chemical potential is higher near the boundary than that in the bulk. The hydrogel finally reaches equilibrium after a long time, e.g., *t*_1_/*t*_0_ = 2 × 10^7^ shown in Figure 6a. When χ is higher, phase separation occurs more easily and faster in the hydrogel. Moreover, more domains are observed during the phase separation process, and the stretch ratio between the swollen and shrunk domains is higher when the hydrogel reaches equilibrium. Figure 6c shows the influence of the system size on the distribution of stretch when the hydrogel reaches equilibrium. The red dashed line is the theoretical prediction of the common tangent construction, while the solid lines are the simulation results, which approach the theoretical prediction when the ratio of the sample length to the characteristic length, *L*/*l*, is large. On the other hand, when *L*/*l* is small, we cannot obtain two phases corresponding to the homogeneous solutions, and the stretch distribution cannot be predicted by the common tangent construction. When L/*l* is small enough, the system is stabilized by the interfacial energy, and phase separation can be suppressed. Figure 6d shows the *L*/*l*–λ_0_ diagram of phase separation at different χ. For given χ, phase separation occurs above the curve, while phase separation does not occur below the curve. Higher χ is required for the occurrence of phase separation of a smaller system.

### 4.3 Phase Separation of Hydrogels under Constrained Biaxial Deformation

**Figure 7:**
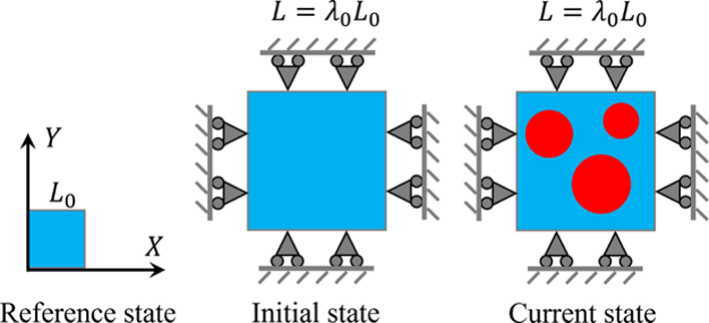
Schematic of a hydrogel under constrained biaxial deformation in the reference, initial, and current states. In the current state, the hydrogel undergoes phase separation with different colors representing the distribution of the concentration of different phases.

We next consider that a hydrogel under constrained biaxial deformation with initial stretch λ_0_ undergoes phase separation (Figure 7). On the boundaries, the hydrogel is assumed to be able to move freely in the parallel direction but have no displacement in the perpendicular direction; i.e., we impose the boundary conditions
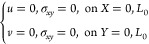
22where *u* and *v* are the displacements of the hydrogel in the *X* and *Y* directions in the current state with respect to the initial state, respectively. It is worth mentioning that large mesh size (≈5*l*) is used for the simulation of the large sample, where the coarse meshes still give a good convergence of results.

**Figure 8:**
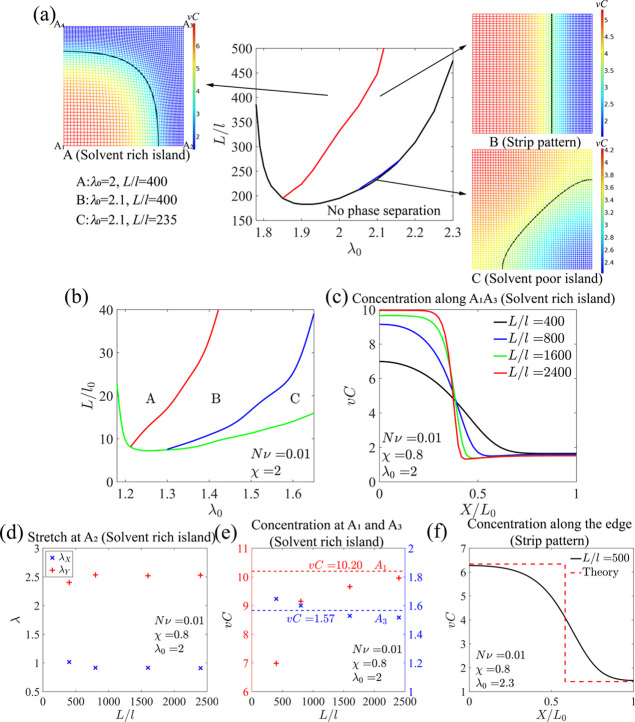
(a) *L*/*l*–λ_0_ phase diagram of phase separation for χ = 0.8. Three patterns, the solvent rich island (λ_0_ = 2, *L*/*l* = 400), strip (λ_0_ = 2.1, *L*/*l* = 400), and solvent poor island (λ_0_ = 2.1, *L*/*l* = 235), are shown in snapshots A, B, and C, where the color bars represent the normalized concentration *vC*, and the black solid curves correspond to the initial concentrations. (b) *L*/*l*–λ_0_ phase diagram of phase separation for χ = 2. (c) Equilibrium distribution of the concentration along the diagonal of the hydrogel in the solvent-rich-island pattern with different sizes of the hydrogel *L*/*l* and the initial stretch λ_0_ = 2. (d) Stretch values in *X* and *Y* directions at corner A_2_ in the solvent-rich-island pattern as functions of the sample size *L*/*l*. (e) Concentrations at corners A_1_ and A_3_ in the solvent-rich-island pattern as functions of the sample size *L*/*l*. The dashed lines are predicted by the common tangent construction. (f) Equilibrium distribution of the concentration along the edge of the hydrogel in the strip pattern.

Figure 8a is the *L*/*l*–λ_0_ diagram of phase separation for χ = 0.8, with the different regions in the phase plane showing no phase separation, and phase separation with different morphologies. The three simulation contour plots demonstrate the distribution of the normalized concentration *vC* for the hydrogel under equilibrium, where the black solid curves correspond to the initial concentrations of the hydrogel, and the mesh grids, which are uniform in the initial state, show the deformation of the hydrogel. Three patterns, the solvent rich island, strip, and solvent poor island, are observed. Since the interfacial energy is proportional to the length of the interface between the swollen and shrunk phases, the length of the interface needs to be minimized to reduce the total energy of the system. Therefore, the solvent-rich-island pattern is expected to occur when the solvent concentration of the hydrogel is relatively low, and the solvent-poor-island pattern occurs when the solvent concentration is relatively high. It is also noted that the deformation of the swollen and shrunk phases in the strip pattern is highly anisotropic. When χ is increased to 2, the boundary between phase separation and no phase separation is significantly lower, indicating that phase separation can occur in much smaller hydrogels (Figure 8b). Moreover, the solvent-poor-island pattern can occur in a much broader region in the phase plane.

We next plot the concentration distribution along the diagonal A_1_A_3_ to characterize the solvent-rich-island pattern for different sample sizes *L*/*l* (Figure 8c). Due to the strain mismatch between the two phases, we see an inhomogeneous distribution of the concentration. When the sample size is large, the concentration distribution along the diagonal A_1_A_3_ is non-monotonic. In the shrunk region, the concentration reaches the minimum close to the interface and slightly increases when the position is away from the interface until it reaches a plateau, indicating a homogeneous shrunk phase. It is noted that the portion of the inhomogeneous region is finite in the shrunk phase (Figure 8c). When the sample size becomes smaller, the concentration distribution is smoothened due to the influence of the interfacial energy. The two phases at corners A_1_ and A_3_ are almost homogeneous and isotropic when the sample size is large enough, while the deformation at corners A_2_ and A_4_ is anisotropic (Figure 8a), which violates the assumption of the constrained biaxial deformation for the homogeneous solutions. The stretch values in *X* direction differ a lot from those in *Y* direction at corner A_2_ for hydrogels with different sample sizes (Figure 8d). The concentrations of the swollen and shrunk phases at corners A_1_ and A_3_ are plotted as functions of the sample size (Figure 8e), where the concentration of the swollen phase shows particularly strong size dependency. The dashed lines are obtained by the common tangent construction for the homogeneous solutions, which agrees well with the simulation results when the sample size is large. The similar size effect on the concentrations of the swollen and shrunk phases is also observed in the solvent-poor-island pattern. Different from the solvent-rich-island and solvent-poor-island patterns, the strip pattern shows a concentration distribution similar to that of the homogeneous solution of the constrained uniaxial deformation, instead of that of the constrained biaxial deformation (Figure 8f), and the deformation gradient can be assumed to be .

## 5 Phase Separation Induced by Quench and Stretch

Since phase separation of a hydrogel is limited by diffusion of solvent molecules, it is expected that the phase behavior of a hydrogel undergoing a phase transition depends on quenching and loading rates. As discussed in Section 3, quenching a hydrogel under a free swelling condition might induce temporary phase separation, while changing the interaction parameter χ slowly induces homogeneous deformation of the hydrogel without phase separation. Similarly, we anticipate and will show that stretching rates can also affect the volume phase transition of hydrogels. Investigating how quenching and stretching rates influence the response of hydrogels can help us explain some experimental phenomena and provide guidance for material and loading designs.

### 5.1 Influence of the Quenching Rate

**Figure 9:**
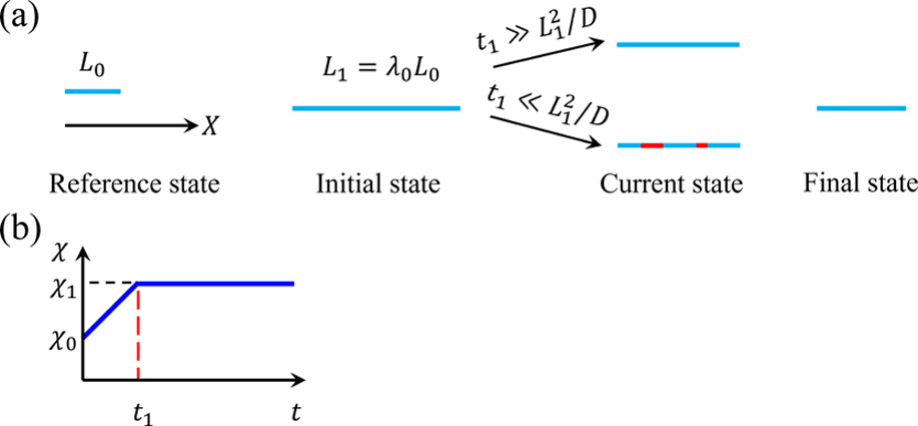
(a) Schematic of a hydrogel under constrained uniaxial deformation with different quenching rates. In the current state, the top schematic represents the hydrogel with a single phase when the loading time is much longer than the diffusion time across the whole sample *t*_1_ ≫ *L*_1_^2^/*D*, and the bottom schematic represents the hydrogel undergoing phase separation when *t*_1_ ≪ *L*_1_^2^/*D*. In both cases, the hydrogel eventually reaches equilibrium and becomes homogeneous (final state). (b) Schematic of the change of χ as a function of time.

Here, we demonstrate the effect of a quenching rate on phase separation using an example of a hydrogel under constrained uniaxial deformation. As Figure 9a shows, the hydrogel is expected to follow different deformation paths depending on the quenching rate. After the hydrogel is allowed to undergo one-dimensionally free swelling in a solvent to reach equilibrium initial stretch λ_0_, the system is quenched by a sudden increase in parameter χ, which induces phase separation (Figure 9a). We consider a linear increase in χ from χ_0_ = 0.5 to χ_1_ = 1.5 within time *t*_1_ prior to holding χ as a constant (Figure 9b). The initial system size is *L*_1_ = 500*l*. Therefore, the diffusion time scale across the whole hydrogel is *L*_1_^2^/*D* = 2.5 × 10^5^*t*_0_, where *t*_0_ is the diffusion time scale across the characteristic length *l*. At the two ends of the hydrogel, we impose the boundary conditions as
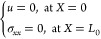
23

**Figure 10:**
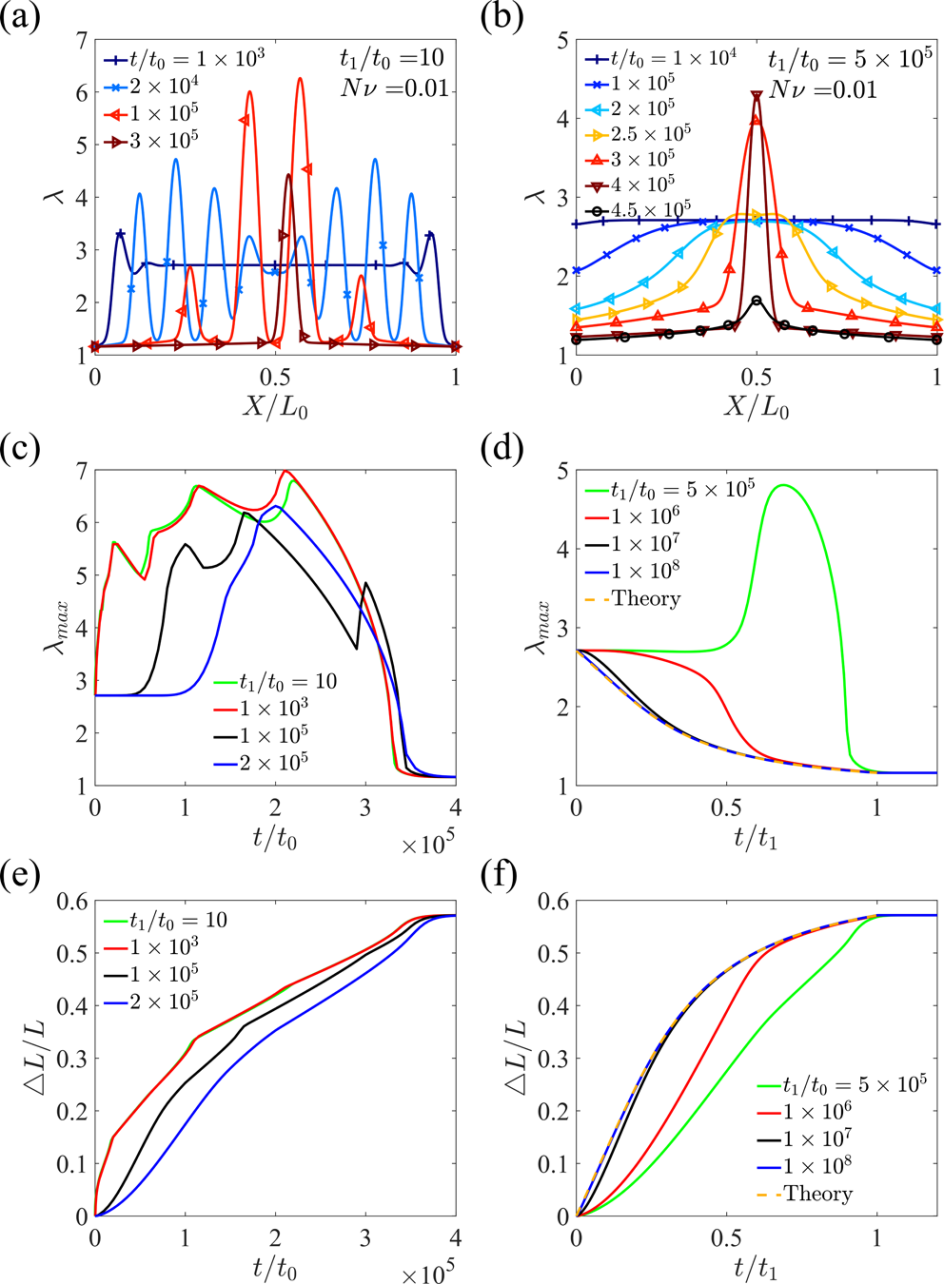
Evolution of stretch distribution along the length of the hydrogel at different times at quenching rates of (a) *t*_1_/*t*_0_ = 10 and (b) *t*_1_/*t*_0_ = 5 × 10^5^. Maximum stretch (c,d) and length change (e,f) as functions of time at different quenching rates.

The evolution of stretch distribution along the length of the hydrogel is plotted at different times for different quenching rates (*t*_1_/*t*_0_ = 10 for Figure 10a and *t*_1_/*t*_0_ = 5 × 10^5^ for Figure 10b), where the solvent diffuses out of the hydrogel through the two ends. When the quenching rate is higher, more domains are observed during the phase separation process. The maximum stretch (Figure 10c,d) and length change (Figure 10e,f) are plotted as functions of time for different quenching rates. Although the length monotonically increases with time, the maximum stretch can non-monotonically increase and decrease with time, attributed to phase separation and the evolution of the swollen phase. The curves are rough at a high quenching rate due to the coarsening of different domains, while the curves are smooth when the quenching rate is low. When the quenching rate is extremely low, namely, *t*_1_ is much larger than the diffusion time across the whole sample (*t*_1_ ≫ *L*_1_^2^/*D*), the hydrogel almost always reaches equilibrium and remains homogeneous with the change of χ (Figure 10d,f), which can be validated by the overlapping of the maximum stretch–time and length change–time curves with those predicted using eq 13. As a result, phase separation does not occur, and the maximum stretch decreases monotonically (Figure 10d). Bai and Suzuki experimentally studied the influence of heating rates on the morphologies of weakly ionized PNIPAM hydrogels,^24^ where the increase in temperature is equivalent to the increase in the parameter χ. They observed that a low heating rate leads to a smooth surface of the hydrogel, while a high heating rate produces a rough surface, which agrees with our simulation results.

### 5.2 Influence of the Stretching Rate

**Figure 11:**
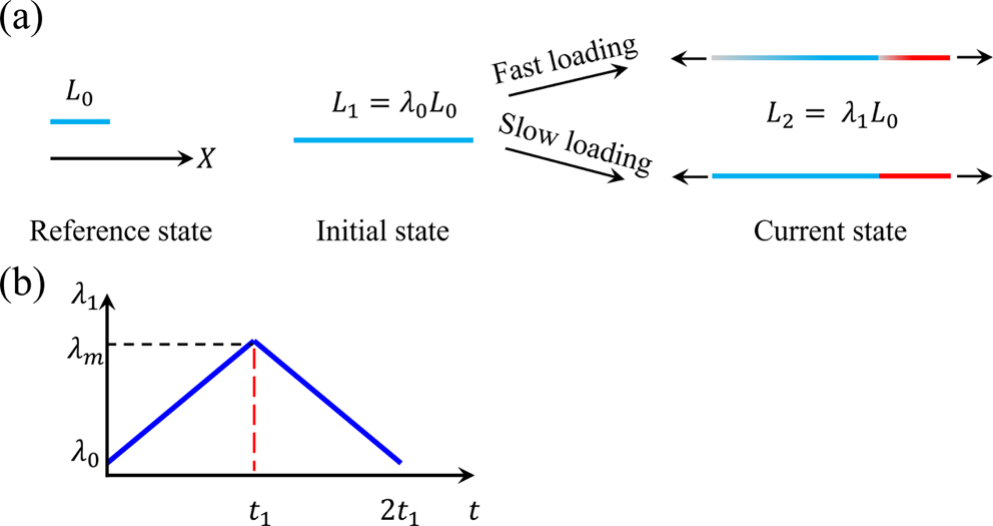
(a) Schematic of a hydrogel undergoing constrained uniaxial stretch in the reference, initial, and current states. In the current state, the top schematic represents the hydrogel with two inhomogeneous phases when the loading is fast, and the bottom schematic represents the hydrogel with two homogeneous phases when the loading is slow. (b) Schematic of the applied stretch as a function of time.

We next study the effect of stretching rates on phase transition. A hydrogel first undergoes one-dimensionally free swelling in a solvent to reach equilibrium initial stretch λ_0_ (Figure 11a). Then, it is gradually stretched at a constant rate from the initial state (*L*_1_ = λ_0_*L*_0_) to the current state with stretch λ_1_ (*L*_2_ = λ_1_*L*_0_) under constant χ (Figure 11). After the stretch reaches λ_1_ = λ_*m*_ at *t* = *t*_1_, it is gradually released to the initial state at the same rate until *t* = 2*t*_1_ (Figure 11b). The total deformation gradient of the hydrogel with respect to the reference state is denoted **F** = diag(λ,1,1).

We consider a hydrogel with χ = 1.5 so that the equilibrium stretch is λ_0_ = 1.16. Then, the hydrogel is stretched to λ_*m*_ = 20 within *t*_1_. We introduce a dimensionless parameter *m* defined as , which characterizes how fast the loading is compared to diffusion across the sample. Upon assuming that the ratio of the initial sample length to the interfacial characteristic length is , we obtain . Here, *n* = 100 is chosen in our simulations.

**Figure 12:**
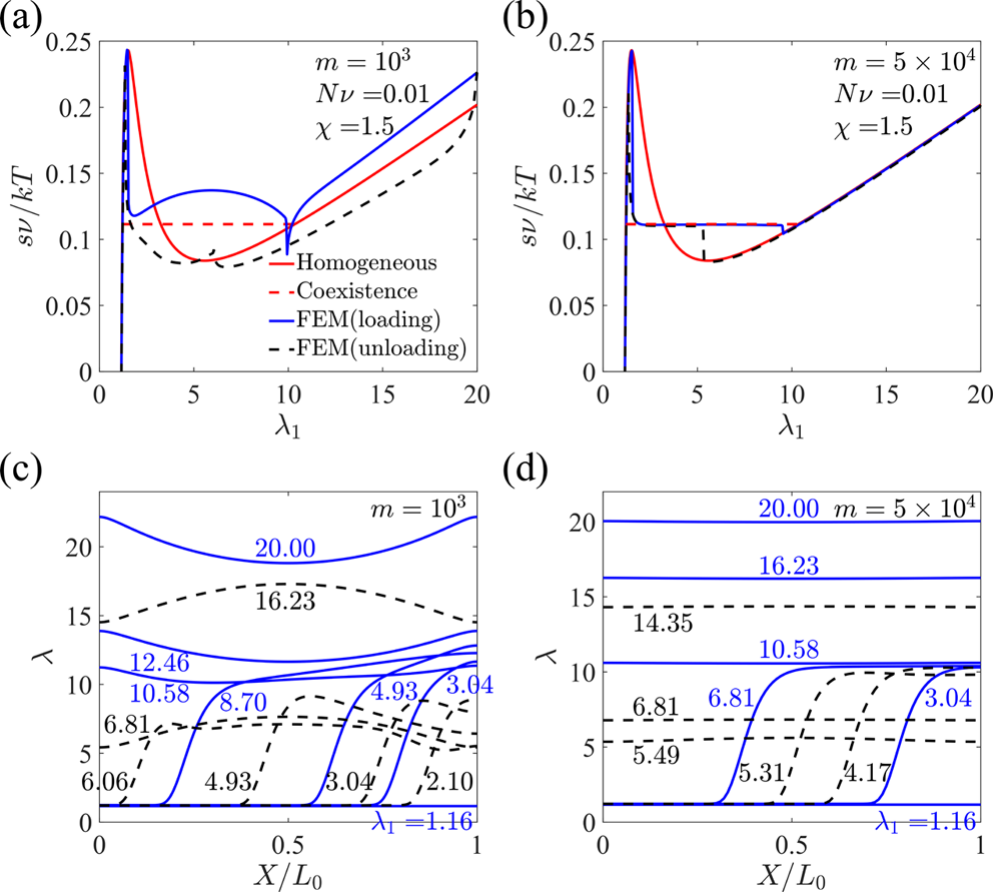
Loading, unloading, and homogeneous equilibrium stress–stretch curves for different ratios of the loading time to the diffusion time across the sample, (a) *m* = 10^3^ and (b) *m* = 5 × 10^4^. The red dashed line represents the stress and critical stretches obtained by the common tangent construction. Stretch distribution at different applied stretches in loading and unloading for (c) *m* = 10^3^ and (d) *m* = 5 × 10^4^, where the solid blue lines correspond to the loading process and the dashed black lines correspond to the unloading process.

The stress–stretch curves are plotted at different stretching rates, where the blue and black curves represent the loading and unloading processes, respectively (Figure 12a,b), while the red solid curve is the homogeneous equilibrium solution obtained from eq 13. As Figure 12a–d shows, the hydrogel remains a shrunk phase when the applied stretch λ_1_ is low. Upon a critical stretch that corresponds to the spinodal point (see Figure 3d, point *c*), the hydrogel becomes unstable, and the swollen phase appears and coexists with the shrunk phase. The shrunk phase gradually transits into the swollen phase as the applied stretch increases. Finally, the hydrogel becomes a single phase again before the applied stretch approaches the stretch value of the binodal point (see Figure 3d, point *b*). The equilibrium stress–stretch relation under the homogeneous assumption obtained in eq 13, where the hydrogel is either in the homogeneous shrunk or swollen phase, is used to compare with the numerical results from the phase-field model. When the loading is fast, the stress deviates from the equilibrium solution from eq 13 with a large hysteresis between loading and unloading (Figure 12a). Higher stress is required to drive the solvent into the hydrogel, while lower stress is needed during unloading. When the loading is slow, the numerical results agree with the theoretical prediction in a wide range of stretch (Figure 12b). A stress plateau is predicted by the numerical solution in both loading and unloading, and it overlaps with the stress obtained by the common tangent construction (the red dashed line in Figure 12b). Figure 12c,d demonstrates the stretch distributions at different applied stretches, where the solid blue lines correspond to the loading process, and the dashed black lines correspond to the unloading process. The results show that a volume phase transition through phase separation is captured by the phase-field model. For a fast loading with *m* = 10^3^, the swollen phase is highly inhomogeneous because it takes time for the diffusion of solvent molecules (Figure 12c). In contrast, the stretch is more homogeneously distributed for a slow loading with *m* = 5 × 10^4^ (Figure 12d). It is worth mentioning that *m* is defined based on the diffusion time scale in the initial state, , instead of in the current state, , which leads to the large value of *m* even for the case of slow loading.

**Figure 13:**
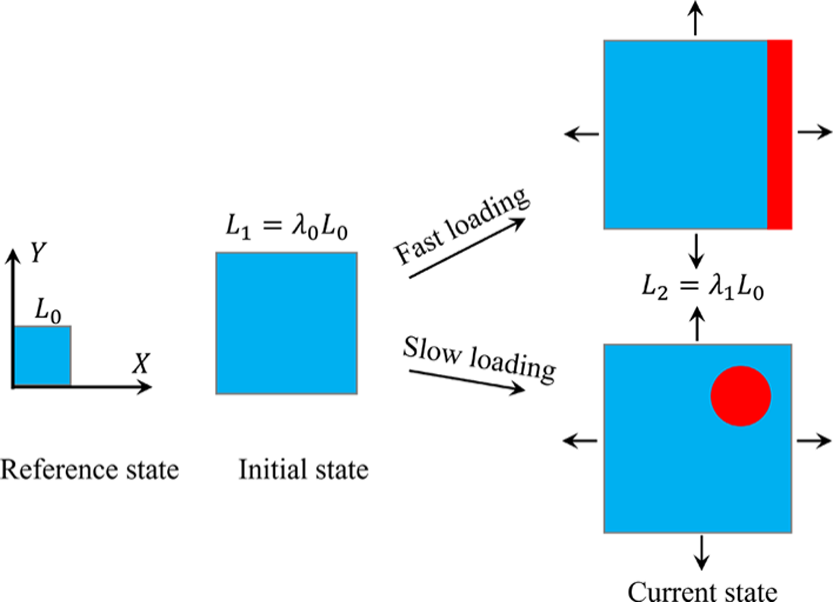
Schematic of a hydrogel undergoing constrained biaxial deformation in the reference, initial, and current states. In the current state, different loading rates lead to different patterns of the hydrogel.

Similarly, a hydrogel undergoing constrained biaxial deformation with the initial stretch λ_0_ at zero stress is gradually stretched equi-biaxially under constant χ from the initial state (*L*_1_ = λ_0_*L*_0_) to the current state with stretch λ_1_ (*L*_2_ = λ_1_*L*_0_) in both *X* and *Y* directions (Figure 13). The initial system size is *L*_1_ = 50*l*.

**Figure 14:**
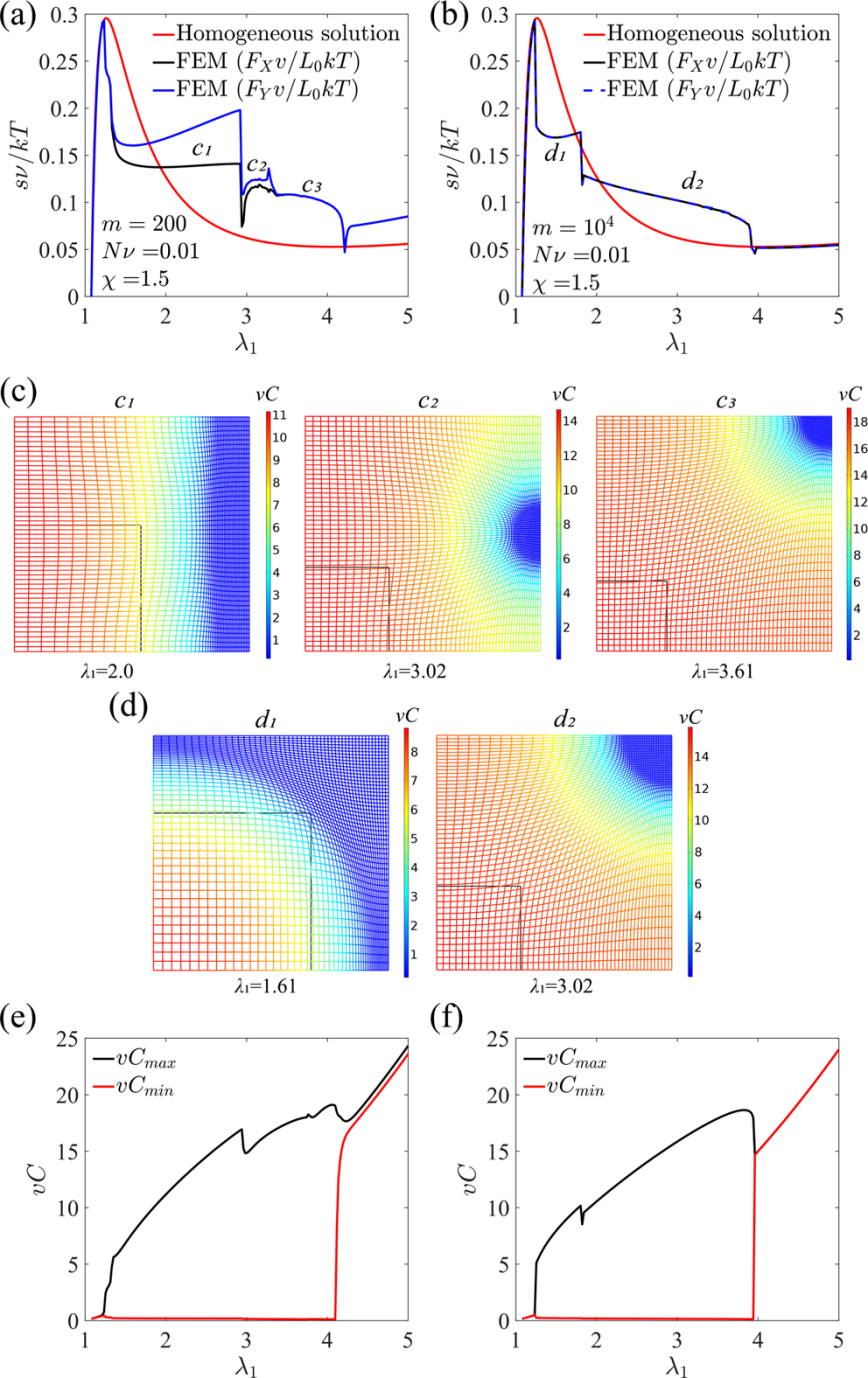
Dependence of the normalized stresses in the *X* and *Y* directions as functions of stretch λ_1_ for the hydrogel subjected to different loading rates (a) *m* = 200 and (b) *m* = 10^4^. Contour plots of the solvent concentration at different stretches for (c) *m* = 200 and (d) *m* = 10^4^. Maximum and minimum solvent concentrations as functions of stretch λ_1_ for (e) *m* = 200 and (f) *m* = 10^4^.

Figure 14a,b shows the stress–stretch curves of the hydrogel at different stretching rates *m* = 200 and *m* = 10^4^, where *m* is defined in the same way as that for the uniaxial case. The average nominal stresses along *X* and *Y* directions defined as *F*_*X*_*v*/*L*_0_*kT* and *F*_*Y*_*v*/*L*_0_*kT*, where *F*_*X*_ and *F*_*Y*_ are the total forces on the surfaces in the *X* and *Y* directions, respectively, are plotted as functions of stretch λ_1_ (Figure 14a,b). The homogeneous stress–stretch solution predicted using eq 7 is also plotted for comparison. During the loading process, the morphology evolves with λ_1_, as shown in Figure 14c,d and Videos S1 and S2, where the black lines represent the boundaries of the initial states. It is noted that different loading rates can lead to different paths of pattern evolution, where the transitions between different patterns lead to sharp changes of stresses (Figure 14a–d). When the loading is fast, the stress obtained from the simulation deviates from the equilibrium solution to eq 7 after phase separation due to inhomogeneous solvent distributions. The stresses in the two directions are not equal, attributed to the occurrence of the strip pattern (*c*_1_), which eventually evolves to the solvent-poor-island pattern (*c*_2_ & *c*_3_) at high enough λ_1_ (Figure 14a,c). In contrast, under a slow loading, the solvent-rich-island pattern (*d*_1_) transits to the solvent-poor-island pattern (*d*_2_) as λ_1_ increases (Figure 14b,d). The morphology is always symmetric with respect to one diagonal, and the stresses in *X* and *Y* directions are equal (Figure 14b). The stress–stretch relations from the simulation overlap with the theoretical prediction when the hydrogel is either completely in the shrunk or swollen phase. The maximum and minimum concentrations of the hydrogel for different loading rates are plotted as functions of λ_1_ for different loading rates (Figure 14e,f). When λ_1_ is larger than a critical value, the hydrogel under slow loading becomes homogeneous again, with the maximum and minimum concentration almost equal (Figure 14f). In contrast, for a fast loading, the maximum concentration is still higher than the minimum concentration when λ_1_ is above the critical value since it takes longer time for the solvent to diffuse into the hydrogel to achieve the equilibrium homogeneous state (Figure 14e).

## 6 Conclusions

In this paper, we reveal the underpinning role of mechanical constraints in triggering the volume phase transitions and phase separation of hydrogels. We show that even for a hydrogel that does not phase-separate during equilibrium free swelling modeled by the Flory–Rehner free energy with a constant interaction parameter χ, multiple phases can coexist when the hydrogel is subjected to mechanical constraints. We obtain the states of equilibrium for the hydrogel under free swelling, hydrostatic loading, constrained uniaxial deformation, and constrained biaxial deformation and unravel how mechanical constraints change the convexity of the free energy and monotonicity of the stress–stretch curves. In contrast to the existence of only one homogeneous solution under equilibrium free swelling, coexistence of multiple solutions, corresponding to multiple phases, can occur in the hydrogel under the above-mentioned mechanical constraints.

By introducing interfacial energy and kinetics of diffusion, we further develop a phase-field model to investigate the pattern evolution in the phase separation of hydrogels under mechanical constraints. For constrained uniaxial deformation, the common tangent construction predicts well the concentrations and fractions of coexisting phases when the hydrogel reaches equilibrium. For constrained biaxial deformation, the two separated phases have large strain misfit, and the homogeneous deformation determined by the common tangent construction is inaccurate, while the concentrations predicted agree quite well with the simulation results when the system size is large enough. As the system size decreases, simulation results deviate from the common tangent predictions. Moreover, the system could be stabilized by the interfacial energy, and phase separation can be suppressed.

We further study the effect of quenching rates and loading rates on the phase behavior of hydrogels. As a result, a quenching rate can significantly influence the pattern evolution of phase separation. Particularly, a fast quenching rate leads to more small domains, resulting in a temporary rough texture of the hydrogel. Although phase coexistence does not occur in a neutral hydrogel under free swelling, fast quenching can trigger temporary phase separation. Similarly, a stretching rate can also influence the pattern evolution of phase separation and stress–stretch responses. A fast loading results in large hysteresis between loading and unloading stress–stretch curves and deviation from the theoretical prediction.

Our theoretical predictions are to be experimentally validated. The effect of mechanical constraints on the phase separation of hydrogels which we predicted is consistent with some reported experiments. Matsuo and Tanaka demonstrated that cylindrical PAAm hydrogels immersed in an acetone solution with different concentrations show different stable patterns with coexistence of swollen and shrunk phases.^32^ Even though PAAm hydrogels undergo continuous volume transitions,^51^ high χ due to the high acetone concentration and the constant volume constraint induced by a dense impermeable skin layer on the surface of the hydrogels lead to phase separation of the hydrogels and morphology changes, consistent with our theory. Regarding the effect of loading rates on phase separation of hydrogels, Bai and Suzuki experimentally observed that heating a weakly ionized PNIPAM hydrogel via a low heating rate leads to a smooth surface of the hydrogel, while a high heating rate produces a rough surface,^24^ consistent with our simulations. However, experimental results that can be quantitatively compared with our theory are lacking. Since our theory is relatively general, we expect that many hydrogels under mechanical constraints can show rich phase behavior. We here call for more experimental studies to quantify the phase separation of hydrogels under various mechanical constraints and loading conditions.

In summary, our study successfully captures rich phase behavior of neutral hydrogels under different mechanical constraints and dynamic loading conditions. It provides design guidelines for hydrogels with a volume phase transition for various applications.

## Supporting Information Available

Videos of simulation results showing phase separation of a hydrogel under constrained biaxial deformation with different loading rates (ZIP)
Phase behavior of hydrogels with χ = χ_1_ + χ_2_ϕ (PDF)
