# Supplementary figures and images for: Mechanics Underpinning Phase Separation of Hydrogels
Source: Macromolecules. 2023 Jan 5;56(2):426–39. doi: 10.1021/acs.macromol.2c02356 (PMC9879212; doi:10.1021/acs.macromol.2c02356)

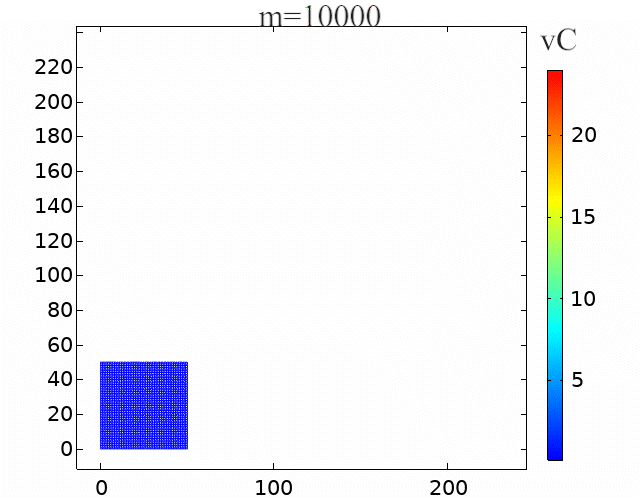

Supplement: Supplementary file 1 — ma2c02356_si_001.zip [file ma2c02356_si_001.zip › simulation videos/Video S2.gif]

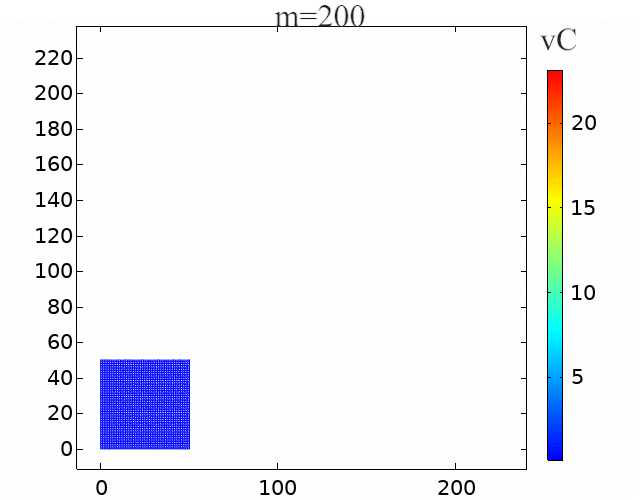

Supplement: Supplementary file 1 — ma2c02356_si_001.zip [file ma2c02356_si_001.zip › simulation videos/Video S1.gif]
