# Supplementary material for: Mechanics Underpinning Phase Separation of Hydrogels
Source: Macromolecules. 2023 Jan 5;56(2):426–39. doi: 10.1021/acs.macromol.2c02356 (PMC9879212; doi:10.1021/acs.macromol.2c02356)
Supplement: Supplementary file 2 — ma2c02356_si_002.pdf [file ma2c02356_si_002.pdf]

Supporting Information

Mechanics underpinning phase separation of hydrogels

Yu Zhou, Lihua Jin*

Department of Mechanical and Aerospace Engineering, University of California, Los Angeles, Los Angeles, CA 90095

*Corresponding author: Lihua Jin

E-mail: lihuajin@seas.ucla.edu

S1. Phase behavior of hydrogels with $\chi = \chi_1 + \chi_2\phi$

The interaction parameters χ for many hydrogels depend on their polymer concentration ϕ. Here we demonstrate that our analysis can be easily extended to different $\chi(\phi)$ functions. A representative linear $\chi - \phi$ relation, $\chi = \chi_1 + \chi_2\phi$ with positive χ_1 and χ_2 constants, is adopted. We study the phase behavior of the hydrogel under free swelling and swelling under hydrostatic loading, and demonstrate that similar to a hydrogel with a constant χ, mechanical constraints can induce phase separation in hydrogels with non-constant χ.

Free swelling

When the hydrogel is under free swelling, we have $s = 0$. Similar to the main text, using the swelling ratio J as a variable, the free energy of the hydrogel is written as

$$W = \frac{1}{2}NkT\left(3J^{2/3} - 3 - 2\ln J\right) + \frac{kT}{v}\left[(J-1)\ln\frac{J-1}{J} + \chi_1\frac{J-1}{J} + \chi_2\frac{J-1}{J^2}\right]. \quad \text{(S1)}$$

When the hydrogel is in equilibrium, we obtain

$$\frac{\partial W}{\partial J} = NkT\left(J^{-\frac{1}{3}} - \frac{1}{J}\right) + \frac{kT}{v}\left[\ln\frac{J-1}{J} + \frac{1}{J} + \frac{\chi_1}{J^2} + \chi_2\left(\frac{2}{J^3} - \frac{1}{J^2}\right)\right] = \frac{\mu_w}{v}. \quad \text{(S2)}$$

The stability of the system is determined by the following equation,

$$\frac{\partial^2 W}{\partial J^2} = NkT\left(-\frac{1}{3}J^{-\frac{4}{3}} + J^{-2}\right) + \frac{kT}{v}\left[\frac{1}{(J-1)J^2} - \frac{2\chi_1}{J^3} + 2\chi_1\left(1 - \frac{3}{J}\right)\frac{1}{J^3}\right] = 0. \quad \text{(S3)}$$

Swelling under hydrostatic loading

We consider a hydrogel under a hydrostatic loading immersed in a solvent with $\mu_w = 0$. The total free energy of the system is

$$G = \frac{d}{2}NkT\left(\lambda^2 - 1 - 2\ln\lambda\right) + \frac{kT}{v}\left[(\lambda^d - 1)\ln\frac{\lambda^d - 1}{\lambda^d} + \chi_1\frac{\lambda^d - 1}{\lambda^d} + \chi_2\frac{\lambda^d - 1}{\lambda^{2d}}\right] - ds\lambda, \quad \text{(S4)}$$

where $d = 3$. The equilibrium condition is rewritten as

$$\frac{\partial G}{\partial \lambda} = dNkT\left(\lambda - \frac{1}{\lambda}\right) + d\frac{kT}{v}\left(\lambda^{d-1}\ln\frac{\lambda^d - 1}{\lambda^d} + \frac{1}{\lambda} + \chi_1\frac{1}{\lambda^{d+1}} - \chi_2\frac{\lambda^d - 2}{\lambda^{2d+1}}\right) - ds = 0. \quad \text{(S5)}$$

The corresponding stability condition becomes

$$\begin{aligned}\frac{\partial^2 G}{\partial \lambda^2} = dNkT\left(1 + \frac{1}{\lambda^2}\right) + d\frac{kT}{v}\Bigg[(d-1)\lambda^{d-2}\ln\frac{\lambda^d - 1}{\lambda^d} + \frac{d\lambda^{d-2}}{\lambda^d - 1} - \frac{1}{\lambda^2} - (d+1)\frac{\chi_1}{\lambda^{d+2}} \\ + \chi_2\frac{1}{\lambda^{d+2}}\left((d+1) - (4d+2)\frac{1}{\lambda^d}\right)\Bigg] = 0\end{aligned} \quad \text{(S6)}$$

We first consider free swelling. The solutions to Eq. (S2) and to Eq. (S3), J as functions of χ_1, for three different χ_2 are plotted in Figs. S1a-S1c, respectively. The equilibrium solutions and the spinodal curves at $\chi_2 = 0.1$ in Fig. S1a show the similar trend to that in Fig. 2b. When $\chi_2 = 0.4$, the spinodal curve in red becomes non-monotonic, where χ_1 first decreases, then increases, and decreases again as the swelling ratio J increases. The black curve of the equilibrium solutions for $\mu_w = 0$ is also always below the spinodal curve in red, and therefore, there is only one equilibrium solution for a given χ_1. The green curve of the equilibrium solutions for $\mu_w = 0.0025kT$ intersects with the spinodal curve at three points, and therefore, there exist two stable equilibrium solutions for certain range of χ_1. The blue curve of the equilibrium solutions when $\mu_w = 0.015kT$ also shows the similar trend with that in Fig. 2b. When $\chi_2 = 0.7$, the black curve of the equilibrium solutions for $\mu_w = 0$ becomes non-monotonic, and there are two points of intersection with the spinodal curve, for χ_1 between which two phases can coexist.

Next, solving Eq. (S6), we show the spinodal curves of a hydrogel subjected to hydrostatic loading, λ as functions of χ_1, at different values of χ_2 in Fig. S1d. The curves with non-zero χ_2 show a similar trend to that with a zero χ_2. Setting $\chi_1 = 0.8$ and solving Eq. (S5), we plot the nominal stress s as a function of λ at different values of χ_2 in Fig. S1e. All the stress-stretch curves are non-monotonic. Though the hydrogel under free swelling may show different phase behaviors at different values of χ_2 (Figs. S1a-S1c), the swelling behaviors are similar when external loading is applied (Figs. S1d-S1e). Therefore, the effects of mechanical constraints on triggering phase separation of hydrogels are carefully studied in the main text for constant χ.

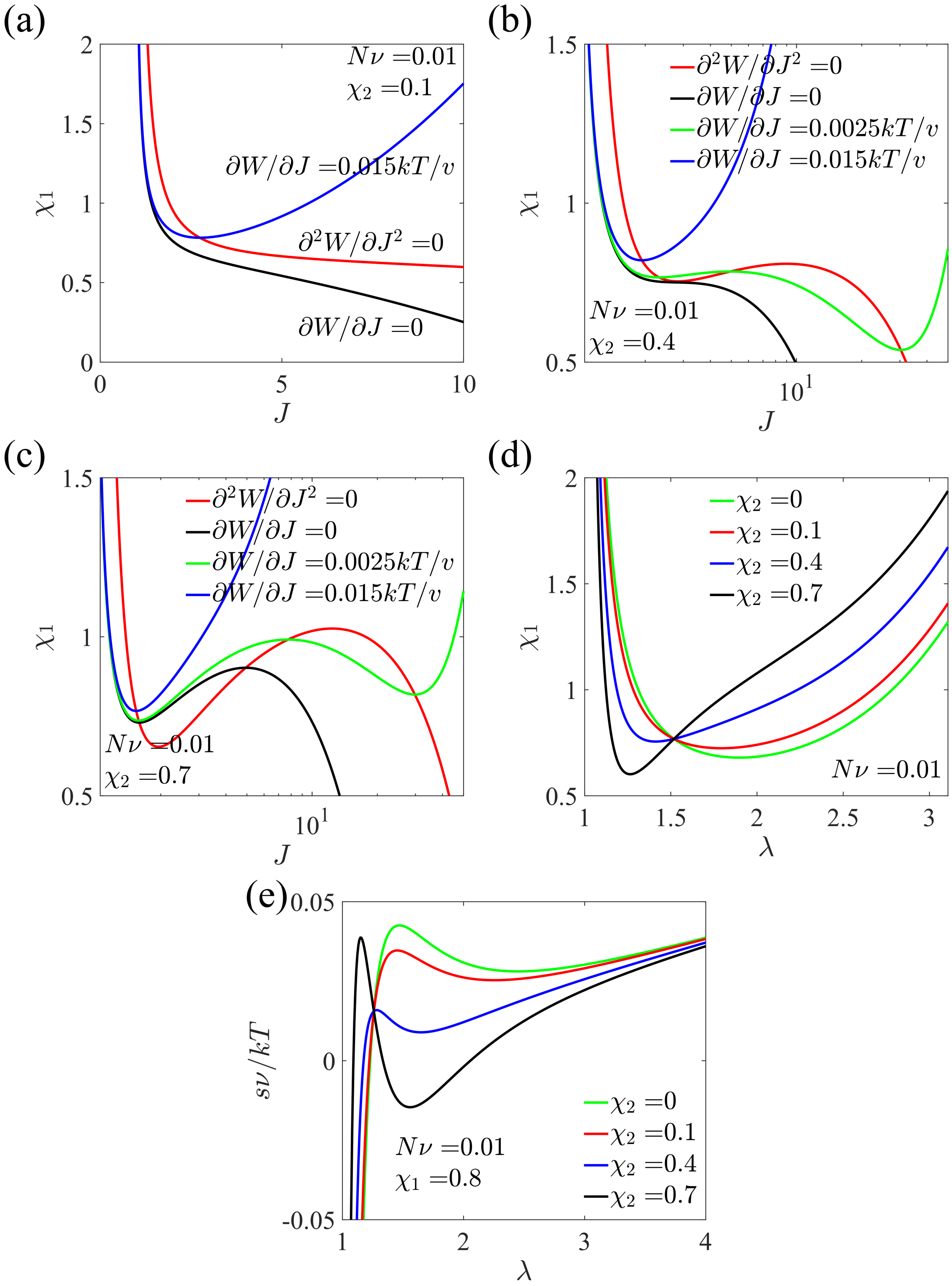

Fig. S1. The equilibrium solutions and the spinodal curves of a hydrogel under equilibrium free swelling at (a) $\chi_2 = 0.1$, (b) $\chi_2 = 0.4$ and (c) $\chi_2 = 0.7$. (d) The spinodal curves of a hydrogel

subjected to hydrostatic loading at various χ_2. (e) Nominal stress as a function of stretch for a hydrogel subjected to hydrostatic loading at various χ_2.

S2. Simulations of phase separation of a hydrogel subjected to constrained biaxial stretch

Video S1. Temporal evolution of solvent concentration showing phase separation of a hydrogel subjected to fast constrained biaxial stretch for $m = 200$ obtained from a phase-field simulation.

Video S2. Temporal evolution of solvent concentration showing phase separation of a hydrogel subjected to slow constrained biaxial stretch for $m = 10^4$ obtained from a phase-field simulation.
